# ‘Moon Shot’: A Case Study of Augmentation of Clozapine With Fluvoxamine in an Adolescent With Treatment-Resistant Schizophrenia

**DOI:** 10.1192/bjo.2024.692

**Published:** 2024-08-01

**Authors:** Lakshmi Keerthana Thatavarthi, Suhas Chandran

**Affiliations:** St John's Medical College and Hospital, Bangalore, India

## Abstract

**Aims:**

Very early onset schizophrenia is considered a rare and severe form of schizophrenia, with onset before age 13. Early initiation of antipsychotics significantly improves outcomes and prognosis. Treatment refractory status is not uncommon and clozapine currently remains the most effective option in this scenario. However, approximately 40–70% of antipsychotic-resistant patients do not respond, or respond only partially to clozapine. Additionally, many patients stop clozapine due to side effects, many of which are due to its active metabolite, nor-clozapine. Since clozapine-resistant patients have limited alternative treatment options, augmentation strategies must be considered.

This case study highlights one such augmentation strategy using fluvoxamine. Fluvoxamine inhibits CYP450 1A2 isoenzymes reducing the risk of the metabolite induced side effects and synchronously increasing plasma concentrations of clozapine.

**Methods:**

The case study is of a 13-year-old female diagnosed with paranoid schizophrenia characterized by second and third person auditory hallucinations, delusions of persecution, paranoid pseudo-community, impulsive aggression and cognitive decline. She screened negative for developmental disorders, metabolic and genetic anomalies and medical co-morbidities. She had failed trials of two atypical antipsychotics. Clozapine was subsequently initiated and optimized to 500 mg/day (Serum Clozapine of 981 mcg/L). Partial improvement in symptomatology was observed. However, dose adjustments were difficult throughout due to side effects of clozapine and pharmacological agents such as Metformin, Lamotrigine, Ipratropium Bromide and Propranolol were used prophylactically to mitigate the side-effects. The polypharmacy, social isolation, excessive sedation and emerging obsessive-compulsive symptoms contributed to secondary negative symptoms. Low-dose fluvoxamine was subsequently used as an augmentation strategy following which improvement was noted.

**Results:**

Several studies have shown that co-administration of fluvoxamine may increase the steady-state serum concentrations of clozapine by a factor of 5. Optimizing the serum ratio of Nor-clozapine and clozapine levels, thereby, reduces the need for aggressive polypharmacy. Low doses of fluvoxamine inhibit the CYT activity, enough to raise the level of clozapine even when the dose of clozapine is reduced by 50% which is the target going forward for this patient.

**Conclusion:**

Although current practice guidelines recommend clozapine mono-therapy for treatment resistant schizophrenia, augmentation of clozapine with fluvoxamine can be considered for those who do not respond adequately to clozapine mono-therapy or cease treatment due to its side effects. However, considering the unpredictable effect on clozapine plasma levels, concomitant use should ideally be initiated in facilities like a pediatric intensive unit where close surveillance is possible especially for side effects such as myocarditis especially in adolescents.

